# Gene Expression Analysis of VEGF and Its Receptors and Assessment of Its Serum Level in Unexplained Recurrent Spontaneous Abortion

**DOI:** 10.22074/cellj.2015.498

**Published:** 2015-01-13

**Authors:** Elham Amirchaghmaghi, Abbas Rezaei, Ashraf Moini, Mohammad Ali Roghaei, Maryam Hafezi, Reza Aflatoonian

**Affiliations:** 1 Department of Immunology, Faculty of Medicine, Isfahan University of Medical Sciences, Isfahan, Iran; 2 Department of Endocrinology and Female Infertility at Reproductive Biomedicine Research Center, Royan Institute for Reproductive Biomedicine, ACECR, Tehran, Iran; 3 Department of Gynecology and Obstetrics, Faculty of Medicine, Isfahan University of Medical Sciences, Isfahan, Iran

**Keywords:** Vascular Endothelial Growth Factor (*VEGF*), Spontaneous Abortion, *VEGF Receptors*

## Abstract

**Objective:**

Unexplained recurrent spontaneous abortion (URSA) is one of the main complications of pregnancy which is usually defined as three or more consecutive pregnancy losses before the 20^th^ week of gestation without a known cause. Vascular endothelial growth factor (VEGF) is a potent angiogenic factor and shown, along with its receptors (VEGFR1, 2), to play important roles in several physiologic processes including reproduction. The aim of the present study was to analyze gene expression of *VEGF* and *VEGF* receptors in endometrium of patients with a history of URSA compared with normal fertile women. In addition, serum *VEGF* concentration was assessed and compared between the two groups at the same time.

**Materials and Methods:**

In this case control study, endometrial and blood samples were obtained between day 19^th^and 24^th^ of menstrual cycle (window of implantation) from 10 women with a history of URSA (case group) and 6 fertile women who had at least one successful pregnancy (control group). Expression of *VEGF* and *VEGFRs* was studied by reverse transcription- polymerase chain reaction (RT-PCR) and then quantified by real time PCR. Normalization of expression levels was done by comparison with beta-actin expression level as an internal control. Relative *VEGF, VEGFR1* and *VEGFR2* expression quantities were compared between the two groups. Enzyme linked immunosorbent assay (ELISA) was used for serum VEGF assay.

**Results:**

*VEGF, VEGFR1* and *VEGFR2* gene expression was detected in endometrial samples of both groups. The mean relative expression of *VEGF* gene was lower in the case group compared with control women, however, both *VEGF receptors* were expressed higher in endometrium of the case group. In addition, the serum level of VEGF was significantly higher in the case group compared with the controls.

**Conclusion:**

Alteration in gene expression of *VEGF* and its receptors in endometrium and changes of serum VEGF might play important roles in pathogenesis of unexplained RSA.

## Introduction

Recurrent spontaneous abortion (RSA), previously known as habitual abortion is usually defined as at least three or more consecutive embryo losses before the 20^th^ week of gestation (1-4). It is estimated that RSA affects 0.5-3% of women in reproductive age (4-6).

Many etiological factors have been considered as cause of RSA including genetic defects such as chromosomal anomalies, anatomic diseases of maternal reproductive tract (congenital or acquired) such as septate uterus, cervical in competence and severe intrauterine adhesions, endocrine abnormalities (including luteal phase deficiency , hypothyroidism, hyperprolactinemia and diabetes mellitus) and immunologic factors such as anti phospholipid antibody syndrome (5, 7, 8). Nevertheless, the cause of RSA remains unknown in around half of the patients despite extensive workup, and thus termed unexplained RSA (URSA) (4, 9). As human endometrium is considered as a fertility determining factor (10), it has been suggested that inappropriate endometrium could be a contributing factor to URSA (11).

On the other hand, vascular endothelial growth factor (VEGF), also known as vascular permeability factor (VPF), is an important angiogenic cytokine. VEGF regulates proliferation, differentiation, and survival of endothelial cells and enhances vascular permeability (12, 13).

VEGF consists of at least six isoforms through alternative splicing in humans (121, 145,165, 183, 189, and 206 amino acids) which have different biological properties and bioavailability (14-16). VEGF functions are mediated via binding to its tyrosin kinase receptors ;VEGF receptor 1 (VEGFR1/ Flt1) and VEGF receptor 2 (VEGFR2/Flk1/KDR). VEGF expression is up-regulated by hypoxia and also different growth factors and cytokines such as epidermal growth factor (EGF), transforming growth factor-β (TGF-β), interleukin-1β (IL-1β) and IL-6 (17).

It has been shown that VEGF plays important regulatory roles in physiological angiogenesis during embryogenesis and reproductive functions (14, 16). Expression of VEGF has been demonstrated in the human endometrium throughout the menstrual cycle with an increase in the late proliferative and secretory phases (18-21). In addition, VEGF expression was found in decidual cells of early pregnancy (21). On the other hand, expression of VEGF receptors was shown in human endometrium (22).

With considering the important functions of VEGF in the reproductive process, some studies focused on the role of VEGF and other angiogenic factors in different female reproductive disorders such as URSA. Lee et al. (23) investigated the polymorphisms of *VEGF* and revealed that *VEGF* polymorphisms and haplotypes are a genetic determinant for the risk of idiopathic RSA in Korean women. Vuorela et al. (24) studied protein expression of VEGF and its receptors in placental and decidual tissues of women with URSA and reported altered expression. Later, Wang et al. (25) showed reduced mRNA and protein expression of VEGF-A in chorionic villi samples of women suffering from URSA. Von Wolff et al. (26) investigated the expression of several cytokines in human endometrium throughout the menstrual cycle by RNase protection assay and also studied 7 URSA patients. They found that mRNA expression of *VEGF* did not significantly change in URSA patients while recently, Lash et al. (27) investigated the expression of seven angiogenic growth factors and their receptors in the different menstrual cycle phases of endometrium from control women as well as in the mid-late secretory phase of women with a history of URSA. They suggested that dysregulation of these factors likely contributes to the etiology of URSA in some cases.On the other hand, Pang et al. (28) studied VEGF and VEGF soluble receptor-1 (sFlt-1) proteins in serum and chorionic villus tissues after abortion in control women (induced abortion) and RSA patients (spontaneous abortion). They revealed a higher expression of VEGF and sFlt-1 in serum and villus tissues of RSA patients who subsequently aborted.

Due to these conflicting reports, we decided to investigate the mRNA expression of *VEGF* and its respective receptors in endometrium of patients with history of URSA compared with normal fertile women in the window of implantation (WOI). In addition, VEGF serum level was simultaneously assessed.

## Materials and Methods

In this case control study, 10 women with a history of URSA who were referred to the infertility clinic of Royan institute were recruited as the case group. Six normal women with proven fertility who were referred to Arash Hospital were considered as the control group. All the cases had been previously evaluated for anatomical, chromosomal, genetic and hormonal abnormalities and had no detectable disorder. None of the studied cases was positive for thrombophilia or abnormal levels of autoantibodies in their serum.

Women with regular menstruation who had at least one successful term pregnancy and were referred for routine gynecologic checkup or who had undergone operations for unrelated procedures such as tubal ligation or tubal re-anastomosis were included in the study as normal controls (29). Control women had no history of abortion or other gynecological disorders.

All subjects signed an informed consent form. This study was approved by the Ethical Committees of Royan Institute and Isfahan University of Medical Sciences. Women were excluded from this study if they were over 40, had any hormonal drug use during the last three months prior to this study or had known systemic, gynecologic or autoimmune disease.

Venous blood and endometrial samples were collected from each woman of both groups between day 19^th^ to 24^th^ of menstrual cycle (WOI) (30, 31). Blood samples were centrifuged at 3000×g for 10 minutes after coagulation .The serum was then collected, aliquoted and stored at -70˚C till use for immunoassay. Endometrial samples were also collected using pipelle (Gynetics Medical Products, Hamont-Achel, Belgium). One piece of each endometrial sample was sent for routine pathologic evaluation and histologic dating was performed according to standard criteria (32). Endometrial samples were cut to pieces of size 5×5 mm and transferred to 2-ml-cryovial tubes (Greiner Bio- One, Frickenhausen, Germany), immediately coated by RNAlater (Ambion, Huntington, UK) and immersed in liquid nitrogen containers for 30 seconds. Finally, the tissue samples were stored at -70˚C until the genomic assay.

### RNA isolation and cDNA synthesis by reversetranscription PCR (RT-PCR)

After thawing the frozen endometrial samples, RNAlater was removed, and then, TRI-Reagent (Sigma, UK) was used for total RNA extraction according to the manufacturer’s instructions as used in our pervious study (33). Total extracted RNA was treated with DNase I (Fermentas, St. Leon- Rot, Germany) to remove genomic DNA contamination. First-strand cDNA was synthesized using oligodT primers and the Superscript II reversetranscriptase system (Fermentas, Germany). Non reverse-transcriptase controls (RT controls) were prepared without adding the enzyme.

The RT-PCR was performed by combining cDNA, Platinum Blue PCR Super Mix (Invitrogen, Paisley, UK) and the forward and reverse primers for *VEGF*, *VEGFR1* and *VEGFR2* (Metabion, Martinsried, Germany). The forward and reverse primer sequences used are shown in table 1. *Beta actin (β-actin)*, a housekeeping gene, was used as internal control. The reaction was continued for 40 cycles under the following thermal conditions: 95˚C for 5 minutes (initial denaturation), followed by 40 cycles of 45 seconds at 95˚C (denaturation), 45 seconds at 60˚C (annealing) and 45 seconds at 72˚C (extention). Negative controls (Nontemplate water instead of cDNA) were also included to ensure lack of reagent DNA contamination. Furthermore, a human placenta sample was used as positive control (34). All PCR products were separated on 1.7% agarose gel (Sigma, UK) by electrophoresis using 1× TAE buffer (Invitrogen, Paisley, UK) and a voltage of 95 V for 40- 50 minutes. Gel documentary machine (Carestream, Berlin, Germany) was used for capture of images.

### Quantitative real-time PCR

Quantitative PCR (Q-PCR) was performed by using 10 μl SYBR Green reagent (Applied Biosystems, USA), 2 μl synthesized cDNA , 2 μl of the same primers that were used in standard PCR (Table 1) and 6 μl of molecular grade water in a total volume of 20 μl. Q-PCR was run in triplicates on ABI StepOnePlus real time PCR machine (Applied Biosystem, Foster, USA). Amplification was performed under the following conditions: 10 minutes at 95˚C, 50 cycles of 95˚C for 15 seconds and 60˚C for 60 seconds. All experiments included negative controls (nontemplate water instead of cDNA). The Q-PCR data were analyzed using the comparative CT method (35). Briefly, the difference in cycle threshold, ΔCT, was determined as the difference between the tested gene and human ß-actin. We then obtained ΔΔCT by finding the difference between the two groups. The fold change (FC) was calculated as 2^-ΔΔCT^.

### Enzyme linked immunosorbent assay (ELISA)

A commercial ELISA kit (Boster Biological Technology, USA) was used according to the manufacturerʼs instructions. In this kit, the serum level of VEGF was assessed by using Biotinylated anti-human VEGF antibody and employing a plate precoated with human VEGF specific monoclonal antibody. Each reaction was carried out in duplicates.

### Statistical analysis

Data analysis was performed using SPSS version 16. Data were compared with t test or Mann-Whitney U test as applicable. Results are presented as mean ± standard error (SE). P value of less than 0.05 was considered as statistically significant.

## Results

In this study, 10 URSA patients and 6 fertile women were enrolled. Demographic and clinical details of participants are presented in table 2. As shown, two groups were matched according to age and body mass index (BMI, p>0.05).

**Table 1:** The sequences, annealing temperatures, and product sizes of the primers used to amplify genes of interest


Primer	Primer sequence (5´-3´)	Annealing temperature	Product size (bp)

**β-actin**	F: CAAGATCATTGCTCCTCCTG	60˚C	90 bp
**β-actin**	R: ATCCACATCTGCTGGAAGG		
**VEGF**	F: TGCAGATTATGCGGATCAAACC	60˚C	81 bp
**VEGF**	R: TGCATTCACATTTGTTGTGCTGTAG		
**VEGFR1**	F: CAGGCCCAGTTTCTGCCATT	60˚C	82 bp
**VEGFR1**	R: TTCCAGCTCAGCGTGGTCGTA		
**VEGFR2**	F: CCAGCAAAAGCAGGGAGTCTGT	60˚C	87 bp
**VEGFR2**	R: TGTCTGTGTCATCGGAGTGATATCC		


β-actin; Beta-actin, VEGF; Vascular endothelial growth factor, VEGFR1; Vascular endothelial growth factor receptor 1 and VEGFR2; Vascular endothelial growth factor receptor 2.

**Table 2:** Demographic and clinical characteristics of URSA and normal women


Variables	URSA women (n=10)	Fertile women (n=6)

**Age (Y)**	30.9 ± 1.24	33.8± 1.10
**BMI (Kg/m^2^)**	25.52 ± 0.99	26.1± 2.11
**Number of previous abortion**	3.8 ± 0.359(range 3-6)	0
**Time of previous abortion (Gestational weeks)**	8.5 ± 1.014(range 5-12)	0
**Parity**	0	1.33± 0.211 (range 1-2)


BMI; Body mass index and URSA; Unexplained recurrent spontaneous abortion.

**Fig 1:**
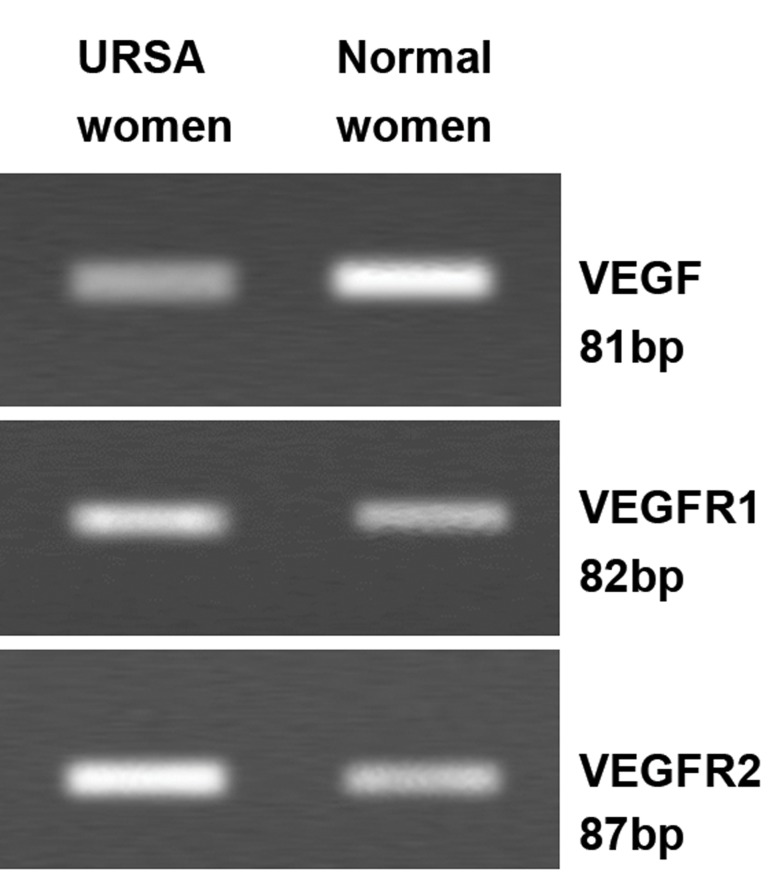
RT-PCR analysis of the expression of VEGF and its receptors in the endometrium of both groups in WOI. RTPCR; Reverse transcription- polymerase chain reaction, WOI; Window of implantation, URSA; Unexplained recurrent spontaneous abortion, VEGF; Vascular endothelial growth factor, VEGFR1; Vascular endothelial growth factor receptor 1 and VEGFR2; Vascular endothelial growth factor receptor 2.

**Fig 2:**
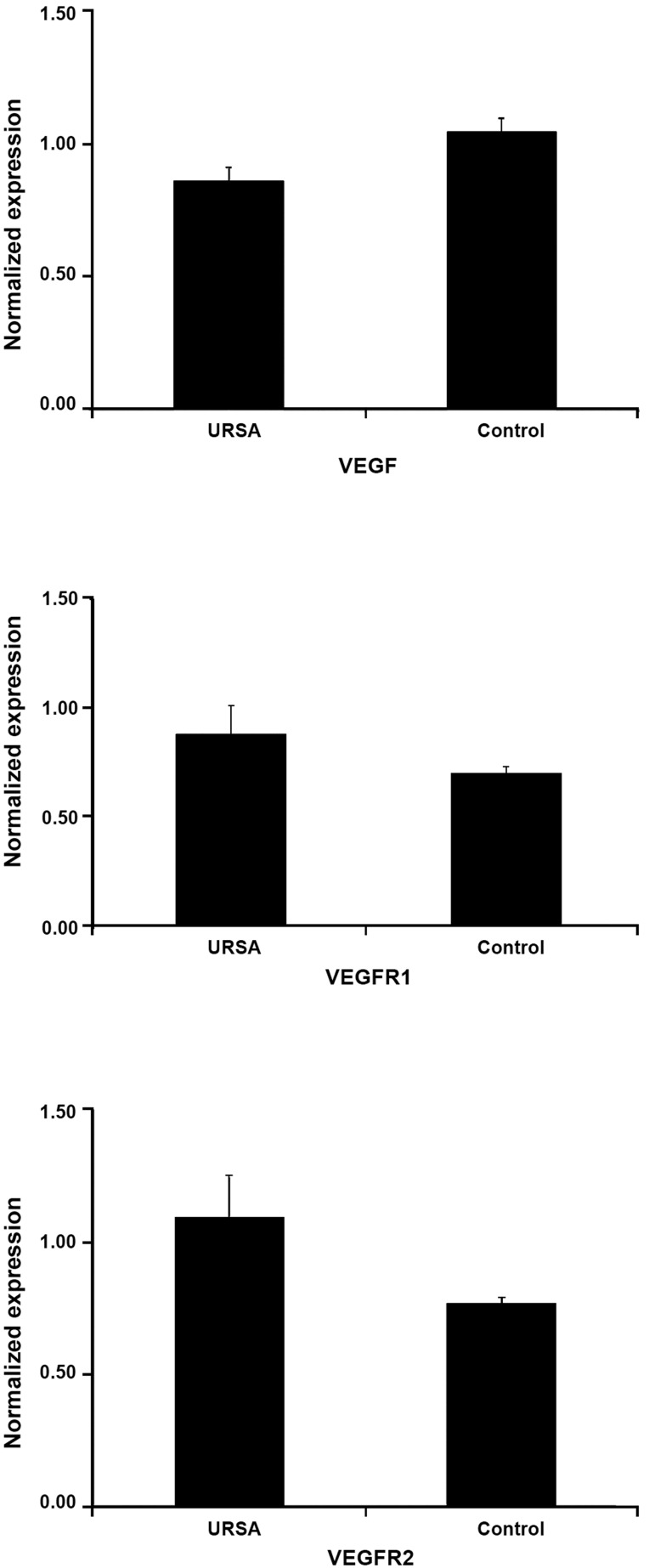
Results of real time PCR for normalized expression of *VEGF, VEGFR1* and *VEGFR2* in endometrium of both groups. URSA; Unexplained recurrent spontaneous abortion, *VEGF*; Vascular endothelial growth factor, *VEGFR1*; Vascular endothelial growth factor receptor 1 and *VEGFR2*; Vascular endothelial growth factor receptor 2.

The results of RT-PCR revealed that *VEGF* and its receptors were expressed in endometrium of both groups in WOI (Fig 1). The real time PCR showed that the mean relative expression of *VEGF* gene was lower in endometrium of women with URSA compared with normal fertile women while both *VEGF receptors* were expressed higher in endometrium of women with URSA (Fig 2). Although these findings were not statistically significant but for VEGF, the calculated p value (p=0.07) was close to significant level.

In addition, the serum level of *VEGF* was significantly higher in URSA group (27.87 ± 7.42 pg/ml) compared with the control group (10.20 ± 2.81 pg/ml) (p=0.044).

## Discussion

The embryo implantation is a fundamental step in reproduction. It needs a suitable communication between the mother and the embryo as a receptive endometrium is a prerequisite (36). Many different factors are suggested to influence endometrial receptivity including different cytokines (37), growth factors (37), matrix metalloproteinase (MMP) (38-39) and adhesion molecules (40).

Among the growth factors, VEGF and its receptors have critical roles in development of the vascular system and processes involved in tissue repair including cyclic renewal of female endometrium (16). Indeed, it was indicated that increased vascular permeability and angiogenesis in uterus are two hallmarks of embryo implantation as VEGF and its receptors are primarily important for uterine vascular permeability and angiogenesis before and during the implantation. In addition, VEGF in complementation with the angiopoietins and their receptor directs angiogenesis during decidualization (41).

In addition, it was shown that disturbances in vascular formation and function may be the contributing factor in different female reproductive disorders such as recurrent miscarriage and implantation failure (42, 43), and unexplained infertility (44).

In the current study, the expression of VEGF and its receptors was investigated in endometrium of women with a history of URSA in the implantation window, i.e. the golden time of embryo implantation. This study revealed that mRNA expression of *VEGF* and its receptors has been altered in comparison with age-and BMI-matched fertile women. As mRNA expression level of *VEGF* was decreased, *VEGF receptors* levels were increased. Although these alterations were not statistically significant but in case of VEGF, the calculated p value was 0.07 which seems it could be significant in larger sample size. In addition, this study showed that VEGF serum concentration was significantly higher in URSA women.

To date, few studies have focused on expression of VEGF and its related receptors simultaneously in endometrium of women suffering from URSA during WOI. von Wolff et al. (26) could not find a significant alteration in mRNA expression of *VEGF* in women with URSA (n=7) compared with fertile controls (n=9) using the RNase protection method. In their study, URSA and control groups were not age matched. In addition, they did not study VEGF receptors. In a later study, Lash et al. (27) investigated expression of several angiogenic factors including VEGF-A and its receptors by IHC and RT-PCR in the endometrium of URSA (n=14) and normal women (n=12) in mid-late secretory phase. Not only they could detect mRNA and protein expression of VEGF and its receptors in endometrium of URSA group but they also showed reduced expression of VEGF and VEGR2 and increased VEGFR1expression in the URSA group. In the latest study, Banerjee et al. (45) found lower VEGF protein expression in endometrium of women with a history of idiopathic RSA during the mid-secretory phase of the menstrual cycle by ELISA. Similar to von Wolff et al. (26), Banerjee et al. (45) did not investigate VEGF receptors. Overall, the findings of the present study were consistent with those of Lash et al. (27) and Banerjee et al. (45). However, there are differences between these studies which include selection criteria of case and control groups (age matched or not, BMI matched or not, fertility status of control group), sample size, ethnic differences, studying mRNA versus protein expression and sensitivity of techniques used (real time PCR versus RNase protection assay and IHC versus ELISA). The increased expression of VEGF receptors in URSA women may be a mechanism compensating for the decreased endometrial VEGF expression but this is only a speculation and needs to be confirmed.

We also observed a higher level of VEGF protein in serum of URSA women in WOI. This finding was in contrast to Al-khateeb et al. (46) and Almawi et al. (47) studies. In both of these studies, a number of *VEGF* polymorphisms were studied in Bahraini women and serum VEGF levels were significantly reduced in RSA women. Although we could not draw any definite conclusion in regard to this difference between these studies,this variation could be due to ethnic differences. As Watson et al. (48) found, some polymorphisms within *VEGF* gene are correlated with variation in VEGF protein production.

## Conclusion

The data of the present study suggest that alteration in expression of VEGF and VEGF receptors is likely to contribute to the etiology of URSA. However, the definite role of VEGF and its receptors in pathogenesis of URSA needs to be further investigated in studies with larger sample size and in more depth including the investigation of their expression in endometrium at the protein level and studying *VEGF* polymorphisms.
